# *In Vitro* Studies of Bacterial Cellulose and Magnetic Nanoparticles Smart Nanocomposites for Efficient Chronic Wounds Healing

**DOI:** 10.1155/2015/195096

**Published:** 2015-05-28

**Authors:** Bianca Galateanu, Mihaela-Cristina Bunea, Paul Stanescu, Eugenia Vasile, Angela Casarica, Horia Iovu, Anca Hermenean, Catalin Zaharia, Marieta Costache

**Affiliations:** ^1^Department of Biochemistry and Molecular Biology, University of Bucharest, 91-95 Splaiul Independentei, 050095 Bucharest, Romania; ^2^Institute of Life Sciences, Vasile Goldis Western University of Arad, 86 Rebreanu, 310414 Arad, Romania; ^3^Advanced Polymer Materials Group, University Politehnica of Bucharest, 1-7 Gh. Polizu Street, 011061 Bucharest, Romania; ^4^Department of Oxide Materials and Nanomaterials, Faculty of Applied Chemistry and Materials Science, University Politehnica of Bucharest, 1-7 Gh. Polizu Street, 011061 Bucharest, Romania; ^5^National Institute for Chemical Pharmaceutical Research and Development, 112 Calea Vitan, 031299 Bucharest, Romania

## Abstract

The quality of life of patients with chronic wounds can be extremely poor and, therefore, over the past decades, great efforts have been made to develop efficient strategies to improve the healing process and the social impact associated with these conditions. Cell based therapy, as a modern tissue engineering strategy, involves the design of 3D cell-scaffold bioconstructs obtained by preseeding drug loaded scaffolds with undifferentiated cells in order to achieve *in situ* functional *de novo* tissue. This paper reports on the development of bionanocomposites based on bacterial cellulose and magnetic nanoparticles (magnetite) for efficient chronic wounds healing. Composites were obtained directly in the cellulose bacterial culture medium by dispersing various amounts of magnetite nanoparticles during the biosynthesis process. After purification and drying, the membranes were characterized by Raman spectroscopy and X-ray diffraction to reveal the presence of magnetite within the bacterial cellulose matrix. Morphological investigation was employed through SEM and TEM analyses on bionanocomposites. The biocompatibility of these innovative materials was studied in relation to human adipose derived stem cells in terms of cellular morphology, viability, and proliferation as well as scaffolds cytotoxic potential.

## 1. Introduction

Skin is the largest organ in the body and, among other critical roles, it serves as an impermeable insulator layer against the environmental microorganisms and prevents dehydration. Loss of skin integrity after injury, surgery, or illness may result in physiologic imbalance and ultimately in significant disability or even death. Wound healing is a complex physiological process that is highly orchestrated by various interrelated factors. The impaired healing of acute cutaneous wounds involves multiple complex pathophysiological mechanisms and is mainly associated with chronic pathologies such as diabetes, cancer, and immunodeficiency [1]. The quality of life of patients with chronic wounds can be extremely poor, thus adding indirect costs to the burden of cutaneous ulcers. Therefore, over the past three decades, great efforts have been made to understand the pathobiology of chronic wounds [2] and to develop efficient strategies to improve the healing process and the social impact associated with these conditions. Consequently, traditional wound healing agents have been largely replaced for chronic wounds by the advanced dressings because topical liquid (povidone-iodine [3]) and semisolid (silver sulphadiazine cream [4] and silver nitrate ointment [5]) formulations do not remain on the wound surface long enough whilst dry traditional dressings (cotton wool, natural or synthetic bandages, and gauzes) do not provide a moist environment for wound healing.

The advantages of tissue-engineered skin replacements (cellular or acellular biometrices) greatly improved the therapeutic potential for chronic wounds. Furthermore, modern strategies in current tissue engineering (TE) applications can provide promising solutions for chronic wounds management. Cell based therapy involves the design of 3D cell-scaffold bioconstructs (cell and drug loaded dressings) obtained by preseeding drug loaded scaffolds with undifferentiated cells in order to achieve* in situ* functional* de novo* tissue [6].

Due to their secretory profile, human adipose derived stem cells (hASCs) delivered into injured or diseased tissue stimulate recovery in a paracrine manner. These cells modulate the “stem cell niche” of the host by stimulating the recruitment of endogenous stem cells to the site of injury and promote their differentiation along the required lineage pathway [7]. hASCs secrete nearly all of the growth factors that take part in normal wound healing process [8, 9]. After implantation, these cells may remain viable at the wound site and secrete growth factors in a continuous and regulated manner in response to environmental cues, just as occurs in the natural wound healing [10].

Furthermore, ideal dermal substitutes should provide a template with appropriate pore structure and mechanical support to guide cells, extracellular matrix (ECM) formation, angiogenesis, and differentiation during the healing process [11].

Cellulose is a linear polymer of *β*(1–4) linked glucose molecules, traditionally sourced from plants. However, refining plant cellulose typically involves harsh, aggressive processing to remove noncellulose materials such as lignin and hemicellulose. Fortunately, bacterial cellulose (BC) represents an attractive alternative as no chemical or mechanical refining is necessary [12–14]. The main production is based on the biosynthesis of cellulose by different microorganisms, including bacteria, algae, and fungi [15–17]. BC differs from plant cellulose with respect to its biocompatibility, purity, high crystallinity, ultrafine network structure, high water absorption capacity, high mechanical strength in the wet state, and availability in an initial wet state [14, 18–21].

Owing to its biocompatibility, BC has recently attracted a great deal of attention for biomedical applications such as artificial skin development or blood vessels replacement [22]. The potential of BC scaffold for* in vitro *and* in vivo* tissue regeneration also continues to be explored and shows great promise for the near future. Moreover, its chemical structure with the presence of hydroxyl bonds provides an excellent hydrophilic matrix for wounds exudate absorption and for the nanoparticles incorporation [22]. BC skin tissue repair biomaterials fabricated by a multilayer fermentation method display a low cytotoxicity and sustain the proliferation of human adipose derived stem cells [23]. Previous studies proved that the presence of BC can promote wound healing by accelerating contractions through the accumulation of extracellular matrix [24]. BC-based biomaterials have been reported to be applied in clinical practice to treat nonhealing foot ulcers [25]. On the basis of fundamental researches on the development of BC biomaterials, some companies have launched several commercial products in wound healing system. BioFill Produtos Biotecnologicos (Curitiba, PR Brazil) developed Biofill and Bioprocess as temporary artificial skin for the treatment of burns and ulcers and Gengiflex for the treatment of periodontal diseases. Xylos Corporation has also developed some BC-based medical devices with dual function of both hydration and absorption properties to maintain the ideal moisture balance [26].

With the rapid development of nanotechnology, magnetic nanoparticles are now being studied all over the world. In recent years, magnetic nanoparticles, especially magnetite, have been used to develop a wide range of biomedical and bioengineering applications such as magnetic resonance imaging [27], contrast agents, biosensors [28], cancer hyperthermia [29], and targeted drug delivery [30]. Most of the envisaged applications are based on the unique magnetic properties of these nanoparticles, namely, their capacity to display high magnetization in the presence of an external magnetic field and insignificant residual magnetism in its absence. The strategies reported to date include polymer-based approaches by loading metal salt precursors into the polymer and inducing* in situ* synthesis of the magnetic nanoparticles [31]. Biopolymers usually show good biocompatibility and subsequently are good candidates for biomedical applications. Hydroxyapatite- (HA-) coated ferrite nanoparticles are one of the most promising materials with rapid magnetization in the presence of a magnetic field [32, 33]. Nanosized magnetic carriers provide good performance due to their higher specific surface area and lower internal diffusion resistance. Based on a recent report, the presence of iron oxide in hydroxyapatite can improve the radiopacity and osteoblast proliferation [34]. Fe_3_O_4_ doped HA exhibits enhanced solubility in physiological solutions compared with HA [35]. Consequently, magnetite nanoparticles loaded biopolymers are promising tools for scaffold mediated construction of 3D engineered tissues composed of magnetic controlled orientation of cells and ECM [36].

In this context, the present paper aims to describe the development of a novel magnetic nanocomposite scaffold made of bacterial cellulose and magnetite nanoparticles for chronic wounds healing in terms of synthesis, complex physicochemical and morphological characterization, and* in vitro* biological behaviour assessment.

## 2. Materials and Methods

### 2.1. Materials

Bacterial cellulose membranes were kindly provided by National Institute for Chemical Pharmaceutical Research and Development (ICCF Bucharest, Romania). The bacterium used in all experiments for obtaining BC was* Acetobacter xylinum DSMZ* (ICCF 398). All chemicals (of analytical grade) were supplied from Sigma-Aldrich and are used as received. Magnetic nanoparticles of Fe_3_O_4_ were obtained directly by coprecipitation using ammonia solution.

The human adipose tissue was obtained from female patients undergoing elective liposuction and all the medical procedures were performed in compliance with the Helsinki Declaration, with the approval of Proestetica Medical Center Committee (reference number 112/23.10.2013). All subjects were in good health and provided written consent before participation in the study. Human adipose derived stem cells (hASCs) were previously isolated from human subcutaneous adipose tissue as described by Galateanu et al. [37]. Briefly, the lipoaspirates (LAs) were processed by collagenase digestion and the cells obtained were resuspended in Dulbecco's modified Eagle's medium (DMEM), supplemented with foetal bovine serum (FBS). The mesenchymal stem cells surface markers panel was confirmed by flow cytometry starting with the 3rd passage. All the cell culture reagents were purchased from Sigma-Aldrich Co. (Steinheim, Germany), while the fluorescent labelling reagents were supplied by Invitrogen, Life Technologies (Foster City, CA).

### 2.2. Methods

#### 2.2.1. Synthesis of Magnetite Nanoparticles (MNP)

Fe_3_O_4_ nanoparticles were prepared by coprecipitating Fe^2+^ and Fe^3+^ ions from corresponding aqueous solution by adding ammonia solution in the presence of polyethylene glycol 200 (PEG200) as soft template, at room temperature. pH of reaction mixture was kept at 12. Fe_3_O_4_ nanoparticles were separated by centrifugation and washed with water and ethanol.

#### 2.2.2. Biosynthesis Process of Bacterial Cellulose—Fe_3_O_4_ Nanocomposites (BC/Magnetite)

Bacterial cellulose (BC) has been obtained as pellicle in National Institute for Chemical Pharmaceutical Research and Development (ICCF Bucharest, Romania) Laboratory from* A. xylinum* DSMZ-2004. The culture medium used for the fermentation of* Acetobacter xylinum* DSMZ-2004 (German Collection of Microorganisms and Cell Cultures) contained an extract from inadequate quality apples and 7.5% glucose, 2% glycerol, 0.2% ammonium sulfate, 0.5% citric acid, and various amounts of magnetite nanoparticles, with the pH being adjusted to 5.5 by acetic acid. The culture media prepared in 500 mL Erlenmeyer flasks were inoculated with 10% (v/v)* A. xylinum* DSMZ-2004 inoculum media and were sterilized by autoclaving at 121°C, for 15 min.

A single* Acetobacter xylinum* colony grown on agar culture medium was transferred to a Petri dish filled with liquid glucose medium and incubated for two days to create a cell suspension. Then, the cell suspension was introduced into the magnetite-dispersed culture medium at 30°C and incubated for 14 days. The magnetite-incorporated BC membrane that was biosynthesized in the medium (*in situ*) was purified by 1 N sodium hydroxide for 2 days at 30°C to remove the cells included in the pellicles. The pellicles were then immersed in water solution of NaN_3_ (0.02%) to reduce microbial contamination neutralized with 1% acetic acid and washed repeatedly with distilled water until its pH was 7.0 and then stored at 4°C. BC/magnetite membranes were obtained with 1, 2, and 5% magnetite content.

#### 2.2.3. Characterization of the Bacterial Cellulose-Magnetite Bionanocomposites

*Physicochemical and Morphological Characterization*.* Raman spectra* of bacterial cellulose, magnetite, and BC/magnetite nanocomposites were recorded on a DXR Raman Microscope (Thermo Scientific) by 633 nm laser line and a number of 10 scans. The 10x objective was used to focus the Raman microscope.

*X-ray diffraction (XRD)* spectra were registered on a Panalytical X'PERT MPD X-ray Diffractometer, in the range 2*θ* = 10–80. An X-ray beam characteristic to Cu K*α* radiation was used (*λ* = 1.5418 Å).

Morphological information including internal structure was obtained through the* scanning electron microscopy (SEM)* analysis of the gold-coated composites. The analysis has been performed using a QUANTA INSPECT F SEM device equipped with a field emission gun (FEG) with a resolution of 1.2 nm and with an X-ray energy dispersive spectrometer (EDS).

Geometrical evaluation (size and shape), crystalline structure of magnetic nanoparticles, and the morphology of the BC and BC/magnetite nanocomposite were investigated by* high-resolution transmission electron microscopy (HR-TEM)* using a TECNAI F30 G^2^ S-TWIN microscope operated at 300 kV with Energy Dispersive X-Ray Analysis (EDAX) facility. Selected area electron diffraction (SAED) for crystalline structure evaluation was also performed.

*Biocompatibility Assessment. Cell Morphology.* The protein expression of actin was studied at 24 h postseeding of hASCs on the magnetic nanocomposites by confocal fluorescence microscopy using a Carl Zeiss LSM710 laser-scanning microscope, with Zeiss 20x and 40x 0.5NA objectives to reveal cellular morphology in direct contact with the biomaterials. hASCs/BC and hASCs/BC/magnetite nanobiohybrids were fixed with 4% paraformaldehyde for 1 h and cell membranes were permeabilized with 2% bovine serum albumin/0.1% Triton X-100 solution at 4°C. Next, the constructs were incubated overnight at 37°C with Alexa Fluor 546 Phalloidin for actin labeling. After cell nuclei were stained with DAPI for 30 min, the resulting labeled nanobiohybrids were inspected in confocal fluorescence microscopy. Carl Zeiss Zen 2010 software version 6.0 was used for image acquisition and analysis.

*Cell Viability.* Live/Dead fluorescence microscopy assay was performed to evaluate hASCs viability in direct contact with the magnetic nanocomposites using the Live/Dead fluorescence based kit. hASCs seeded on BC and BC/magnetite nanocomposites were labelled with calcein AM and ethidium bromide to yield two-color discrimination of the population of live cells from the dead-cell population. In this view, cell-biomaterials bioconstructs were incubated with the staining solution prepared according to manufacturer's instructions, for 15 minutes in the dark. Next, the stained biohybrids were analyzed by confocal fluorescence microscopy using a Carl Zeiss LSM710 laser-scanning microscope and images were captured with Carl Zeiss Zen 2010 software version 6.0.

Subsequently, hASCs viability in contact with the magnetic nanocomposites was quantitatively determined using MTT (Thiazolyl Blue Tetrazolium Bromide) spectrophotometric assay at 24 h and 5 days postseeding. In this context, all cell-biomaterial bioconstructs were incubated in 1 mg/mL MTT solution for 4 hours. The resulted formazan crystals were subjected to solubilisation in isopropanol for 30 minutes at room temperature. The absorbance of the resulting solution was measured by spectrophotometry at 550 nm (Appliskan Thermo Scientific, Waltham, MA, USA). The optical densities obtained are proportional to cell viability. 

*Cytotoxic Potential.* The cytotoxic potential of the studied magnetic nanocomposites on the hASCs was evaluated using “*in vitro* toxicology assay kit lactate dehydrogenase based” according to the manufacturer's protocol. Briefly, the culture media were harvested at 24 h and 5 days postseeding and they were mixed with the solutions provided in the kit. After 20 minutes of incubation at room temperature and darkness, the LDH concentration was determined by measuring the optic density of the resulting solutions at 490 nm (Appliskan Thermo Scientific, Waltham, MA, USA). 

*Statistical Analysis.* The spectrophotometric data were statistically analysed using GraphPad Prism 3.03 software, one-way ANOVA, and Bonferroni test. The experiments were performed with *n* = 3 biological replicates and each data set is presented as the average of three replicates (mean ± standard deviation).

## 3. Results and Discussions

### 3.1. Raman Analysis

**Figure 1:**
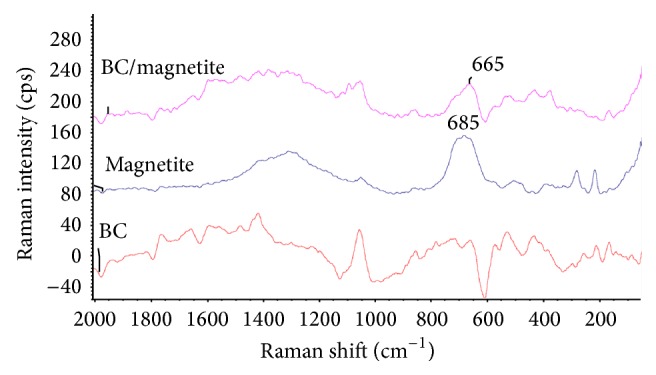
Raman spectra of pure BC, magnetite, and BC/magnetite 5% membranes.

The Raman spectra of BC, magnetite, and the corresponding BC/magnetite nanocomposites are shown in Figure 1. BC displays bands in the 250–550 cm^−1^ range which, according to the literature, can be assigned to C–O, C–C–C, C–O–C, O–C–O, and C–C–O deformation. The absorptions around 1090–1100 cm^−1^ could be associated to the stretching mode of C–C and C–O bonds. Pure magnetite displays a single peak at around 660–685 cm^−1^ which is characteristic of the magnetite form of the iron mineral [38, 39]. The BC/magnetite membranes also display the typical peak of magnetite at around 665 cm^−1^ and also other peaks in the range of 200–350 cm^−1^. Some of these peaks could be assigned to the BC component in the membrane.

### 3.2. X-Ray Diffraction Analysis

**Figure 2:**
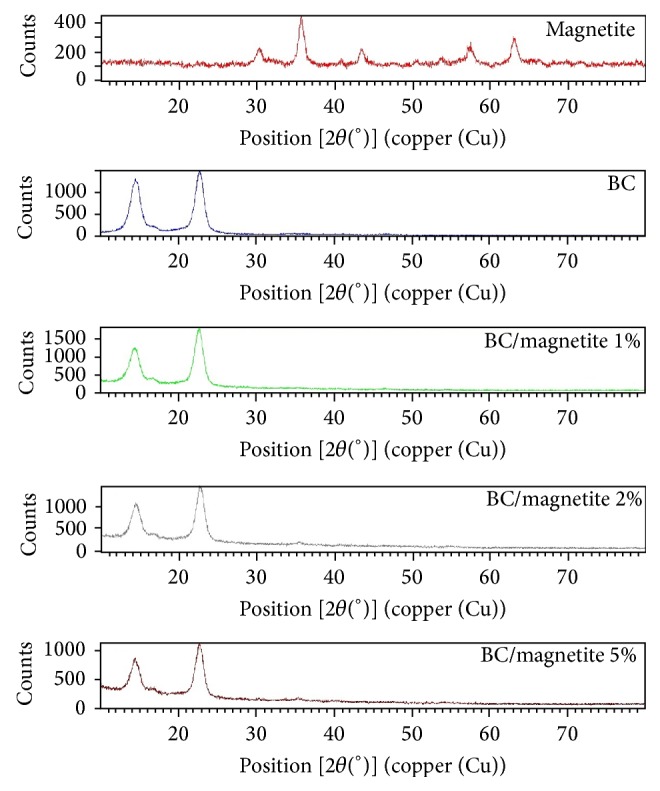
XRD diffractograms of pure BC, magnetite, and BC/magnetite composites.

XRD-diffraction was also employed to investigate the crystalline structure of the BC/magnetite bionanocomposites (Figure 2).

The high crystallinity degree of cellulose was associated with 3 maxima of diffraction peaks at 2*θ* = 14.6°, 16.95°, and 22.97°. The XRD pattern of the pure magnetite presents the peaks at 2*θ* = 30.27°, 35.7°, 43.44°, and 57.14°, which are indexed to (220), (311), (400), and (511) for Fe_3_O_4_ (magnetite with face-centered cubic system). Diffractograms of BC/magnetite composites reveal similar maxima to pure cellulose and magnetite, especially at 35.7° ((311) index for magnetite) slightly shifted according to nanocomposite composition: 35.49° for BC/magnetite 1%, 35.63 for BC/magnetite 2%, and 35.56° for BC/magnetite 5%.

### 3.3. SEM Morphology

**Figure 3:**
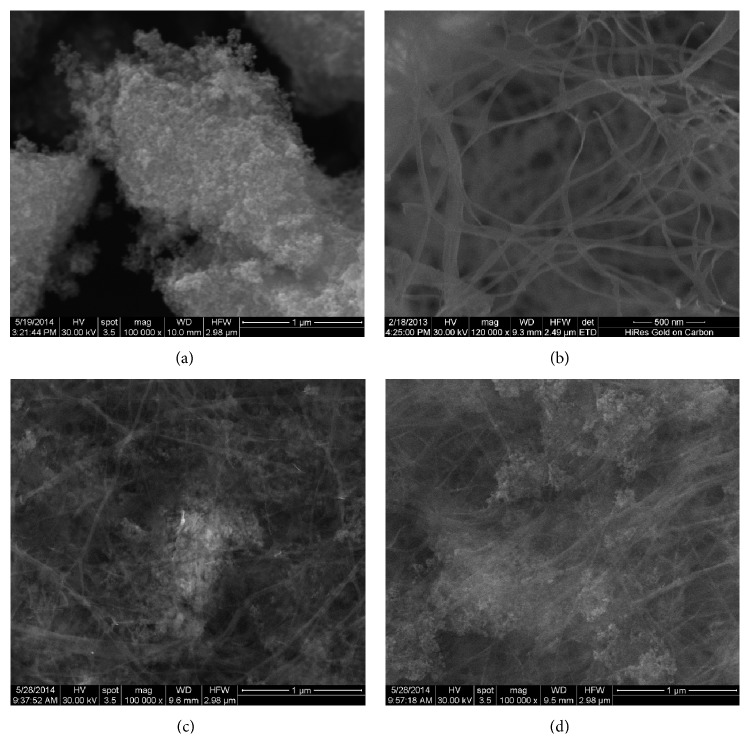
SEM microphotographs of pure magnetite nanoparticles (a), bacterial cellulose (b), nanocomposite with 1% magnetite (c), and 5% magnetite (d).

Micro- and nanostructural characteristics of magnetic membranes of bacterial cellulose and magnetite were revealed by SEM analysis.  Figure 3 shows the nanostructure of the components (magnetite in image (a) and bacterial cellulose in image (b)). (c) and (d) images reveal the nanostructure of bionanocomposites with various concentrations of magnetite. The presence of magnetite onto the surface or within the cellulose nanofibres can be noticed. At high magnetite concentration (5%), a more uniform covering of the nanofibre is observed.

### 3.4. TEM and SAED Investigation

**Figure 4:**
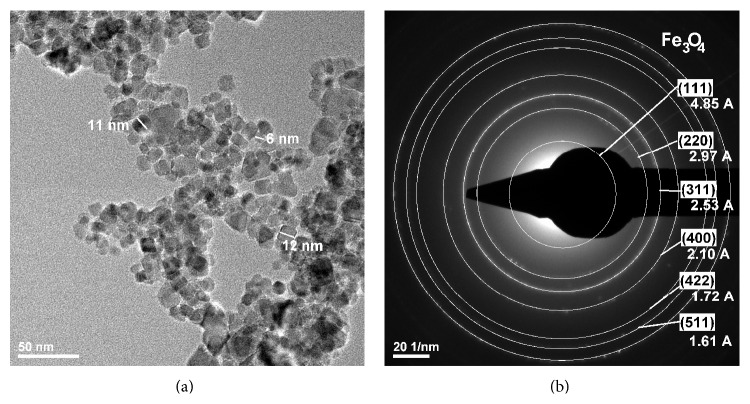
TEM and SAED images of pure magnetite nanoparticles.

To better show the presence of magnetite within the bacterial cellulose membranes TEM analysis was employed for the bionanocomposites in comparison with pure cellulose and magnetite powder. Pure magnetite appears as nanoparticles with average diameter of 6-7 nm (Figure 4(a)). Selected electron diffraction (SAED) analysis (Figure 4(b)) associated with the nanozone from Figure 4(a) indicates a nanocrystalline sample with clear indexing for Fe_3_O_4_ as cubic crystalline network with centred faces (in accordance with ICDD file number 04-008-8146).

**Figure 5:**
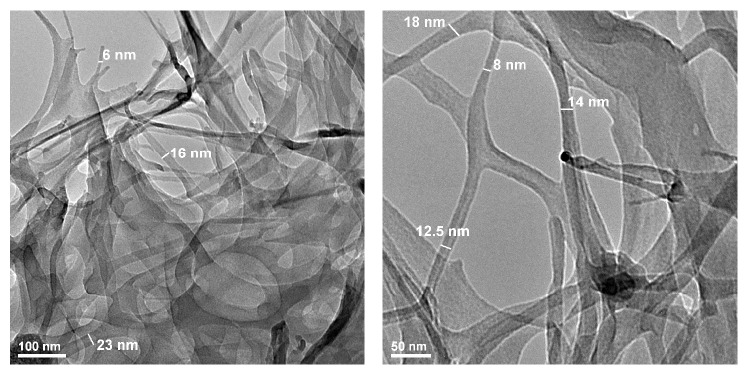
TEM microphotographs of bacterial cellulose.

Bacterial cellulose has a three-dimensional nanofibrillar structure (as already shown by SEM analysis) with an average fibre diameter of 20 nm, clearly revealed by the bright field TEM investigation (TEM-BF, Figure 5).

**Figure 6:**
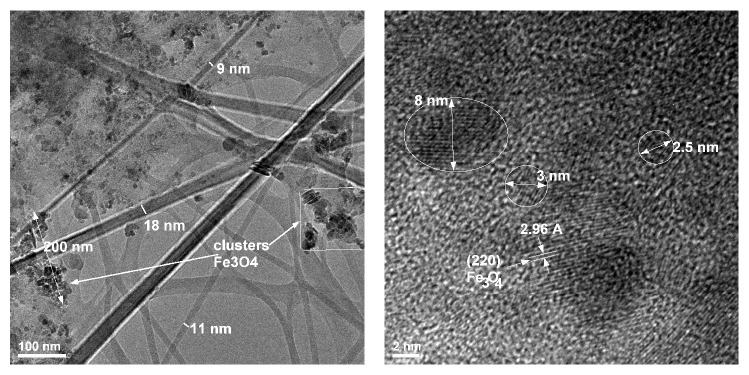
TEM and HR-TEM images of BC/magnetite membranes with 5% MNP content.

TEM investigation of the BC/magnetite bionanocomposites offers valuable information on the composites nanostructure, localization, and dispersion of magnetite nanoparticles within the BC fibrillar structure. Therefore, this study shows that the composite membranes have individual well-dispersed magnetic nanoparticles and magnetite clusters or aggregates onto and within the BC nanofibres. Even at the lowest concentration of magnetite (1%) in bacterial cellulose, one may notice individual magnetic particles of 3–5 nm embedded in the nanofibre. MNP clusters of 17–30 nm could be also seen onto the surfaces of BC fibres. At higher magnetite concentrations (5%), the tendency towards agglomerations and bigger clusters of MNP is present. The magnetic nanoparticles clusters are chained thus forming microblocks. Nanosized clusters and individual magnetic nanoparticles are also present within the BC nanofibres (Figure 6). Fe_3_O_4_ particles with 3–8 nm diameter are also seen, while the crystalline planes are supported by Miller indexes (220) with interplanar distance of 2.6 Å.

### 3.5. hASCs Morphology on BC/Magnetite Nanocomposites

**Figure 7:**
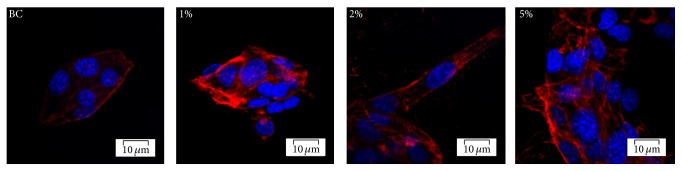
Confocal fluorescence microscopy micrographs of hASCs actin filaments network (red fluorescence) in hASCs/BC, hASCs/BC/magnetite 1%, hASCs/BC/magnetite 2%, and hASCs/BC/magnetite 5%, DAPI-stained nuclei are blue.

hASCs morphology on BC and BC/magnetite nanocomposites and their ability to interact with the substrate material in terms of adhesion and cytoskeleton development were investigated after 24 h of culture. As shown in Figure 7, hASCs displayed long and distinctive actin filaments surrounding the nuclei on all magnetic nanocomposites, which clearly determined cell overall morphology.

Actin microfilaments formation may be assigned with a shape modelling process that occurs in response to the direct contact of the cell with the biomaterial. The actin cytoskeleton underlies the cell adhesion process, which is highly important for further tissue formation. Additionally, a higher cell density was observed for BC/magnetite 5% than the other magnetic nanocomposites or pure BC.

### 3.6. hASCs Viability in Contact with BC/Magnetite Nanocomposites

#### 3.6.1. Live/Dead Fluorescent Microscopy Assay

**Figure 8:**
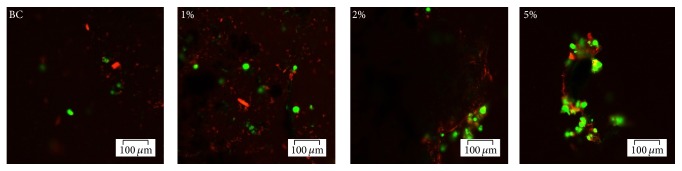
Confocal fluorescence microscopy micrographs revealing live and dead cells on BC, BC/magnetite 1%, BC/magnetite 2%, and BC/magnetite 5%, after 24 h of culture.

In order to examine cell survival on the tested biomaterials, the viability of hASCs was evaluated at 24 h postseeding by confocal fluorescence microscopy (Figure 8), based on the simultaneous staining of live (green labelled) and dead (red labelled) cells.

After 24 h postseeding, bright green labeled cells were observed on the surface of all tested biomaterials but in a greater amount on BC/magnetite 5% nanocomposite as compared to the other tested compositions. Furthermore, the Live/Dead confocal micrographs confirmed the higher cell density on BC/magnetite 5% nanocomposite as compared to the BC control and BC/magnetite 1% and BC/magnetite 2% nanocomposites. The ratio between the green (living) and red (dead) cells was constant in all the tested samples.

#### 3.6.2. MTT Assay

**Figure 9:**
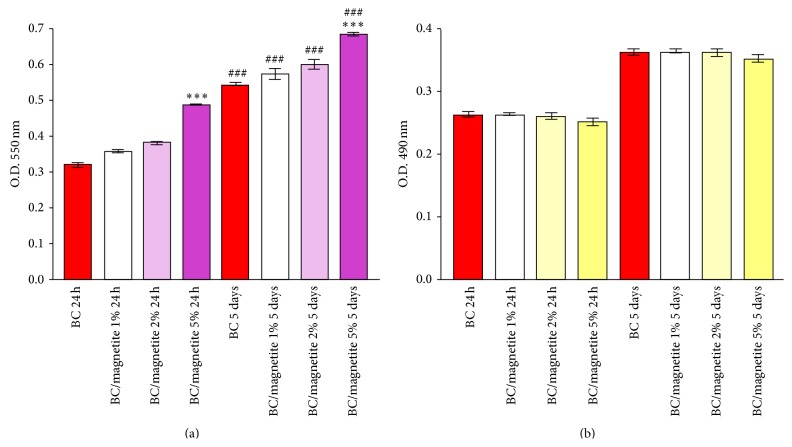
Quantification of (a) hASCs viability on pure BC, BC/magnetite 1%, BC/magnetite 2%, and BC/magnetite 5% nanocomposites, as revealed by MTT test at 24 h and 5 days postseeding. [^*∗∗∗*^ *p* < 0.001 (hASCs/BC versus hASCs/BC/magnetite 5% nanocomposites) and ^###^ *p* < 0.001 (hASCs/BC 24 h versus hASCs/BC 5 days; hASCs/BC/magnetite 1% 24 h versus hASCs/BC/magnetite 1% 5 days; hASCs/BC/magnetite 2% 24 h versus hASCs/BC/magnetite 2% 5 days; hASCs/BC/magnetite 5% 24 h versus hASCs/BC/magnetite 5% 5 days)] and (b) pure BC, BC/magnetite 1%, BC/magnetite 2%, and BC/magnetite 5% nanocomposites' cytotoxic potential effect on hASCs after 24 h and 5 days of culture as revealed by LDH assay.

hASCs viability on pure BC and BC/magnetite nanocomposites was quantitatively determined by MTT spectrophotometric assay. In this context, the hASCs/BC and hASCs/BC/magnetite 1%, hASCs/BC/magnetite 2%, and hASCs/BC/magnetite 5% biohybrids were subjected to MTT spectrophotometric assay at 24 h and 5 days of culture and the results are represented in Figure 9(a).

Our results showed that hASCs survived on all tested magnetic nanocomposites after 24 h of culture. Significant differences were detected in terms of cell viability between the pure BC and BC/magnetite 5% nanocomposite (*p* < 0.001), confirming the fluorescent microscopy observations regarding the cell density displayed on BC/magnetite 5%. Additionally, all the BC-based nanocomposites sustained hASCs proliferation. After 5 days of culture, significantly increased (*p* < 0.001) values of cell viability were detected in all bioconstructs as compared to 24 h of culture.

### 3.7. Biomaterials' Cytotoxic Potential on hASCs

The cytotoxic potential of BC/magnetite 1%, BC/magnetite 2%, and BC/magnetite 5% was evaluated by spectrophotometric quantification of the LDH enzyme release in the culture media by hASCs seeded in direct contact with the samples. Similar to the MTT assay, a pure BC membrane served as reference for this experiment and the results are represented in Figure 9(b). Spectrophotometric data revealed constant values of LDH activity in all the tested biomaterials suggesting that none of the samples induced cell toxicity. No statistical significant differences were detected after data analysis.

## 4. Conclusions

Nanocomposites based on bacterial cellulose-magnetic nanoparticles were developed in this study by direct introduction of the MNP within the cellulose culture medium. At higher concentrations of magnetite in the cellulose membranes, the magnetic nanoparticles are more uniformly dispersed, covering a higher surface of the bacterial cellulose nanofibres. There are also some areas where the covering of magnetite was not so uniform, resulting probably from the initial dispersion within the BC culture medium. The obtaining of such a bionanocomposite directly from the cellulose culture medium is an efficient technique, while the biosynthesis process is not affected by the presence of magnetite nanoparticles.

Fluorescent labelling of actin filaments showed that hASCs displayed a normal morphology in contact with all the tested magnetic nanocomposites. Microscopic detection of both living and dead cells showed that hASCs survived after 24 h of culture in contact with all tested biomaterials. Additionally, no significant differences were displayed in the ratio between live and dead cells. However, the MTT spectrophotometric assay revealed significant differences between the pure BC (reference) and BC/magnetite 5% in terms of cellular viability at 24 h and 5 days postseeding, whereas LDH assay results showed that none of the tested samples exerted cytotoxic effects on hASCs after 24 h. Nevertheless, all the BC-based nanocomposites promote hASCs proliferation, a key feature in the wound healing process.

In conclusion, due to its increased physical, chemical, morphological, and biological properties as compared to pure BC, BC/magnetite 5% nanocomposite could be considered for further* in vivo* wound healing studies.
